# Massive Osteolysis and Pseudotumor Formation following Maestro Total Wrist Arthroplasty

**DOI:** 10.1155/2024/1301778

**Published:** 2024-03-04

**Authors:** Marcus Sagerfors, Daniel Reiser

**Affiliations:** Department of Orthopedics and Hand Surgery, Faculty of Medicine and Health, Örebro University, SE 70182 Örebro, Sweden

## Abstract

Metallosis is a known complication of arthroplasty and has been reported for the hip, knee, and shoulder joints. Metallosis pseudotumors have been linked to an increased risk of implant failure. We report a case of pseudotumor with massive bone loss following total wrist arthroplasty (TWA) using the Maestro implant. Revision to arthrodesis is possible, but issues with bone loss have to be addressed. We recommend caution in offering TWA to young patients with high functional demands.

## 1. Introduction

Total wrist arthroplasty (TWA) is a motion-preserving option in the treatment of wrist arthritis. Despite advances in implant design over the years and improved survival rates, a recent randomized controlled trial comparing two modern implant designs found that roughly one-third had undergone reoperations during the first two years postoperatively [1]. Metallosis is a known complication of arthroplasty and has been reported for the hip, knee, and shoulder joints. Periprosthetic osteolysis (PPO) is a known complication after TWA. One contributing cause may be small particle disease, triggering a complex immunologic reaction resulting in osteoresorption and osteolysis. However, a paper by Boeckstyns et al. found no correlation between polyethylene debris and PPO [2].

Metallosis and pseudotumor development have been reported after using the Universal 2/Freedom TWA (Integra Life Sciences Corporation, Cincinnati, OH, USA) in up to 20% of cases, and metallosis pseudotumors have been linked to an increased risk of implant failure [3, 4]. The Universal 2/Freedom device has an articulation consisting of a metal radial component articulating against a convex ultra-high-molecular-weight polyethylene (UHMWPE) bearing which is locked to the carpal plate and thus interchangeable. The Maestro TWA (Biomet, Warsaw, Indiana, USA, now discontinued) uses a metal convex carpal component articulating with a concave UHMWPE radial articulating surface, which is compression molded onto a cobalt-chrome alloy radial body with a stem of titanium. In the case of polyethylene wear, exchanging just the polyethylene component is not possible. To our knowledge, there are no reports on the Maestro TWA and pseudotumors.

## 2. Case Presentation

**Figure 1:**
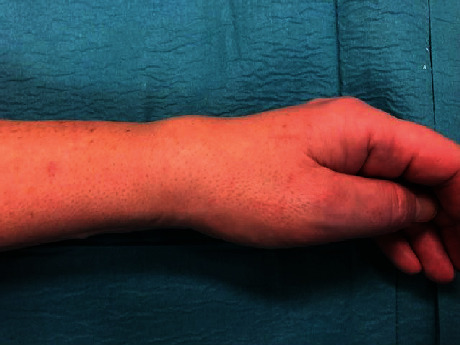
Preoperative swelling of the wrist.

**Figure 2:**
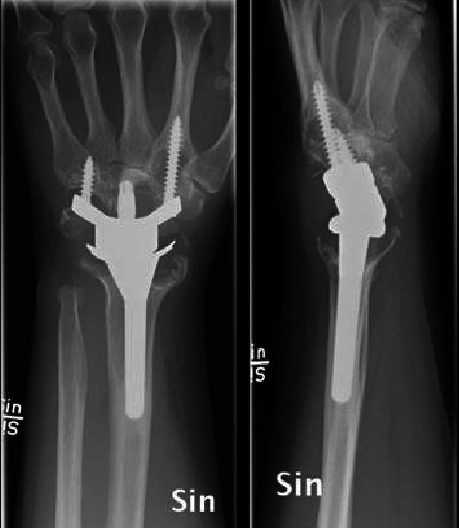
Preoperative radiographs with substantial osteolysis.

**Figure 3:**
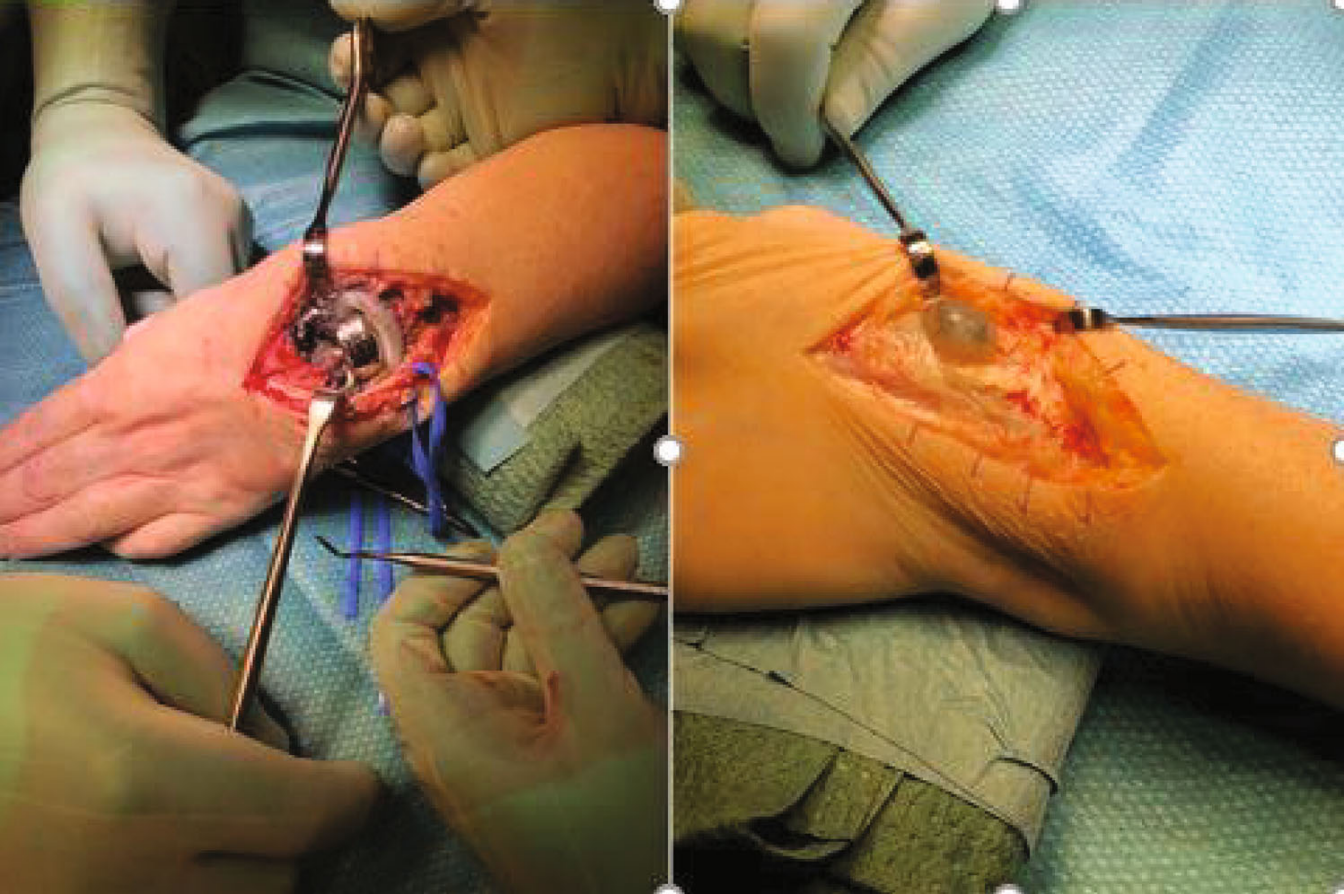
Intraoperative findings with black metallosis and synovitis.

**Figure 4:**
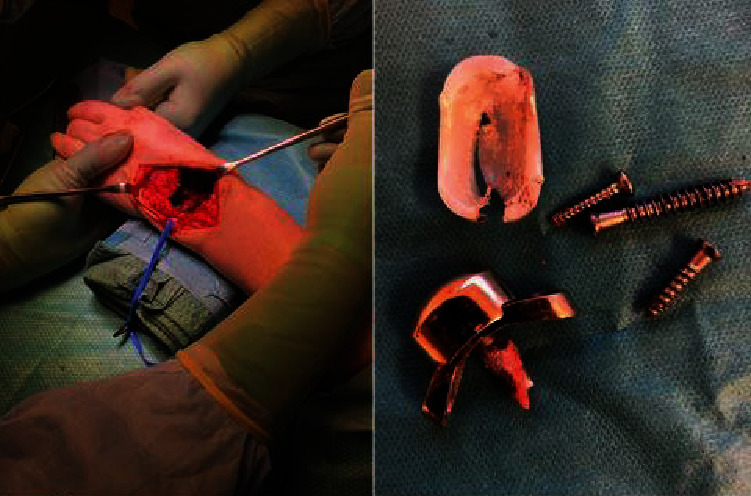
Wear of the polyethylene and substantial bone loss.

**Figure 5:**
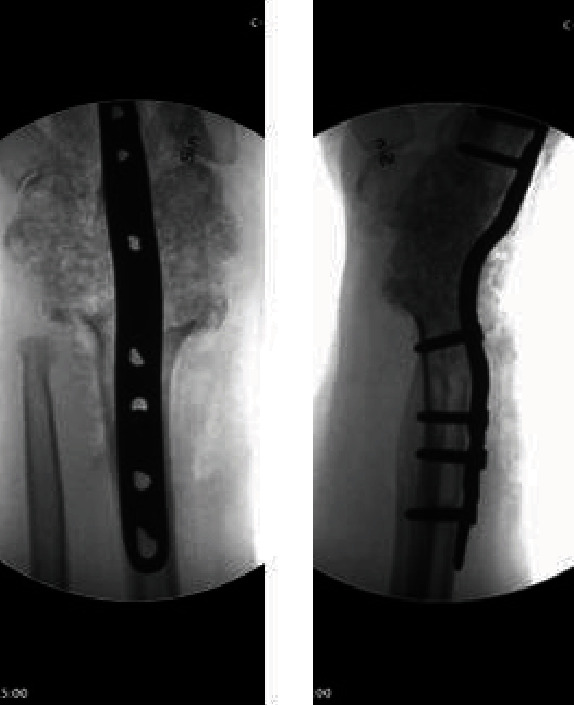
Intraoperative radiographs after bone grafting and dorsal plate fixation.

**Figure 6:**
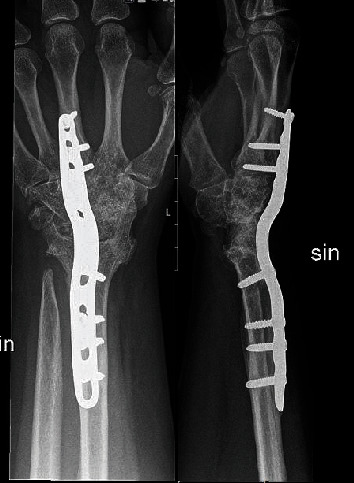
Radiographs 3 years postoperatively with bony union.

Here, we present a case of pseudotumor formation 10 years after primary TWA surgery using the Maestro implant. The patient was a 49-year-old woman with rheumatoid arthritis working as an assistant nurse who presented with increasing pain in her left wrist. She was under medication with paracetamol, methotrexate, NSAIDs, and injections with TNF-*α* inhibitor (adalimumab). She had used her left wrist without any limitations after the initial TWA operation. Clinically, there was considerable swelling at the dorsoradial aspect of the wrist but no signs of infection (Figure 1). Her rheumatic disease was well controlled, but no formal grading of the disease activity was done [5]. Preoperative hand grip strength in the left wrist was 10 kilograms (kgs) compared to 34 kgs on the right side. Regarding patient-reported outcome measures (PROMs), the preoperative Patient-Rated Wrist Evaluation score (PRWE) was 83, and the Disabilities of the Arm, Shoulder and Hand score (DASH) was 68. Radiographs showed osteolysis around both the radial and carpal components with almost complete destruction of the capitate (Figure 2). After discussion with the patient, it was decided to revise to an arthrodesis. As the patient is working as a nurse assistant including some heavy lifting, the position of the arthrodesis was discussed, and the patient opted for a wrist in slight extension to ensure maximum grip power. It has been shown that a wrist in 30° extension provides maximum grip strength without compromising wrist endurance [6]. During the operation, black metallosis was noted, and substantial wear of the radial polyethylene articulation resulted in metal-on-metal contact between the carpal and radial components (Figures 3 and 4). Intraoperatively, there were no signs of infection, and tissue cultures were not taken. The bony defect after implant removal was filled with cancellous bone from the femoral head allograft, and fixation was done using a locking plate (Synthes DePuy, West Chester, PA) (Figure 5). Radiographs, 10 weeks postoperatively, demonstrated bony union. At the latest follow-up 3 years postoperatively, the quick-DASH score was 6.8, the PRWE-score was 3, and the hand grip strength was 20 kgs on the left and 28 kgs on the right side. Radiographs showed bony union (Figure 6). VAS pain scores (0-10 where 10 represents the worst pain imaginable) at rest and during activity were both 0. Pronation was 90° for the right side and 70° for the left side, and supination was 65° for the right side and 80° for the left side. The patient is still working full time as an assistant nurse.

## 3. Discussion

TWA is a well-established procedure, but despite advances, reoperations are frequent [1]. As wrist motion is preserved, it can be a feasible option in selected patients with wrist arthritis [7]. Pseudotumor formation due to metallosis is rare but may arise after TWA. The cause of pseudotumors is unclear but has been linked to increased wear [4]. There have been arguments that the osteoclastic effect caused by metallic debris is responsible for both PPO and implant loosening. Metallosis pseudotumors increase the risk of implant failure, which in some cases can be asymptomatic or associated with little pain [3]. Revision to arthrodesis after failed TWA is possible, but severe bone loss may be an issue [8]. A previous study of failed TWA in rheumatoid patients using tricortical iliac crest bone graft and Steinmann pins found that conversion to an arthrodesis is possible, but complication rates are high [9]. Conversion of a Maestro TWA to wrist arthrodesis after postoperative infection has been reported using a femoral head allograft [10]. In our patient, there was substantial bone loss of the capitate, but the carpal peg was still firmly attached to the remaining capitate at the time of revision surgery. The polyethylene articulation of the proximal component demonstrated extensive wear. This may be related to her using the wrist for lifting and heavy loading without any limitations. There have been speculations whether younger patients who maintain a large range of motion can have eccentric loading of the polyethylene component leading to accelerated wear [11]. However, it has been shown previously that polyethylene wear does not correlate with PPO [2]. At presentation, our patient had severe wrist dysfunction with a decreased grip strength and poor function assessed by the DASH and PRWE scores. Due to the massive bone loss, a revision to another TWA was considered unsuitable. Following revision to arthrodesis, a stable and pain-free wrist was achieved with good PROMs and grip strength. We used a cancellous femoral head allograft, but another option has been described using a decorticated femoral head allograft where the femoral neck is contoured and tapered by a sagittal saw and high-speed burr to fit the medullary defect in the radius [12].

## 4. Conclusion

Given the substantial bone loss associated with metallosis and pseudotumor formation, we recommend caution in offering TWA to young, high-demand patients.

## Data Availability

Data supporting this research article are available from the corresponding author or first author on reasonable request.
